# Photosynthetic usable energy explains vertical patterns of biodiversity in zooxanthellate corals

**DOI:** 10.1038/s41598-022-25094-5

**Published:** 2022-12-02

**Authors:** Tomás López-Londoño, Kelly Gómez-Campo, Xavier Hernández-Pech, Susana Enríquez, Roberto Iglesias-Prieto

**Affiliations:** 1 Department of Biology, Pennsylvania State University, University Park, PA 16802 USA; 2 Unidad Académica de Sistemas Arrecifales, Instituto de Ciencias del Mar y Limnología, Universidad Nacional Autónoma de México, 77500 Cancún, Quintana Roo México

**Keywords:** Ecology, Community ecology, Ecophysiology, Physiology

## Abstract

The biodiversity in coral reef ecosystems is distributed heterogeneously across spatial and temporal scales, being commonly influenced by biogeographic factors, habitat area and disturbance frequency. A potential association between gradients of usable energy and biodiversity patterns has received little empirical support in these ecosystems. Here, we analyzed the productivity and biodiversity variation over depth gradients in symbiotic coral communities, whose members rely on the energy translocated by photosynthetic algal symbionts (zooxanthellae). Using a mechanistic model we explored the association between the depth-dependent variation in photosynthetic usable energy to corals and gradients of species diversity, comparing reefs with contrasting water clarity and biodiversity patterns across global hotspots of marine biodiversity. The productivity-biodiversity model explained between 64 and 95% of the depth-related variation in coral species richness, indicating that much of the variation in species richness with depth is driven by changes in the fractional contribution of photosynthetically fixed energy by the zooxanthellae. These results suggest a fundamental role of solar energy availability and photosynthetic production in explaining global-scale patterns of coral biodiversity and community structure along depth gradients. Accordingly, the maintenance of water optical quality in coral reefs is fundamental to protect coral biodiversity and prevent reef degradation.

## Introduction

Sunlight is the major source of energy for virtually the entire biochemical production of organic matter on Earth^1^. Among the different factors affecting primary producers both in terrestrial and aquatic ecosystems, sunlight is perhaps the most spatially and temporally heterogeneous, resulting in significant impacts on the energy transfer across trophic levels^2–4^. The variation in the supply of usable energy derived from primary production plays an important role on the spatial variation of species diversity in ecological communities, facilitating a decreased risk of species extinction from demographic and environmental stochasticity in highly productive environments^5^. Although a positive productivity-biodiversity relationship prevails in terrestrial and aquatic ecosystems^6^, significant debate remains over the strength of the linkage between these two features and whether it is predictable, considering the observed variations in the form of productivity-biodiversity relationship in some communities (e.g., positive, negative, unimodal and neutral)^7–10^. Since the amount of usable energy by a group of organisms in a community is difficult to measure directly, part of this variation has been associated with the surrogates used as roughly indicative of productivity and the confounding effects of environmental stressors, typically more accentuated in highly productive environments^6,7^.

Scleractinian corals are metazoans with the ability to calcify, responsible for building the most diverse and productive marine ecosystem from which humans obtain essential socio-economic benefits. The ecological and evolutionary success of these animals in oligotrophic environments since the Late Triassic is attributed to the nutritional endosymbiosis with photosynthetic unicellular algae (zooxanthellae)^11^, which confers on them the capacity to use sunlight as the major source of energy increasing their rates of calcification^12^. Due to the exponential attenuation of sunlight with depth^13^, the vertical distribution of symbiotic corals occurs along steep gradients governed by light in small spatial scales, while other limiting resources and physical factors (e.g., nitrogen, oxygen, temperature) vary less or remain nearly constant^14,15^. As benthic, sessile organisms prevalent in coastal waters, corals are also exposed to significant changes in light intensity in response to the optical properties of the water column (i.e., vertical attenuation coefficient for downwelling irradiance, *K*_d_). This variation is determined by precipitation patterns and seaward fluxes of terrestrial nutrients and sediments^13,16^, having important implications on the primary production and energy balance of coral communities^3,4^. However, associations between gradients of productivity and biodiversity in coral communities have not yet been demonstrated, and patterns of coral biodiversity are primarily attributed to biogeographic factors, habitat area and disturbance frequency^10,17–22^.

The obligatory nature of the endosymbiosis between a primary producer and an animal, together with the vertical distribution of corals along strong gradients of sunlight energy, make coral communities particularly interesting for exploring the relationship between primary productivity and consumer-species diversity. Using a mechanistic model based on underwater optics and physiological principles, we estimated the variation in photosynthetic usable energy supplied by the symbiotic algae to coral hosts and examined its association with patterns of coral species diversity across depth gradients. We further tested the model using published datasets from reefs with contrasting evolutionary histories, environmental conditions, and patterns of coral species diversity, encompassing the three major hotspots of global marine biodiversity. Our analysis shows that much of the variation in coral diversity with depth is explained by changes in photosynthetic usable energy, providing support to a mechanistic link between productivity and biodiversity in coral communities.

## Results

### Zooxanthellae’ energy budgets are variable and nonlinearly related to light availability

**Figure 1:**
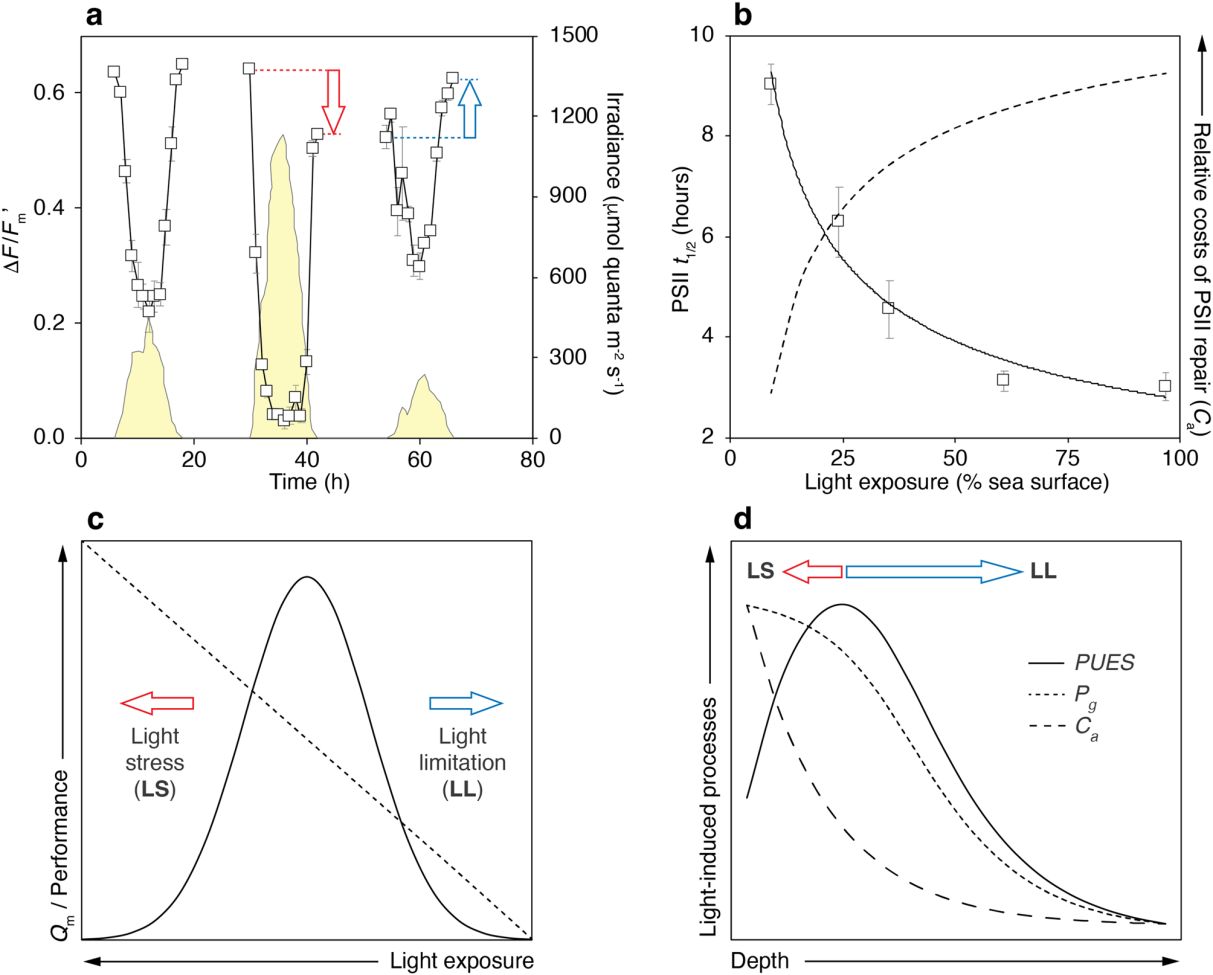
Principles and components of the coral productivity-biodiversity model. (**a**) Diurnal variation of Δ*F*/*F*_m_’ in symbionts of *Porites astreoides* measured experimentally during a control-reference day (first day), and during days with increased (second day) and reduced light exposure (third day), emphasizing the consequences on the *F*_v_/*F*_m_ recovery at the end of the diurnal cycle (arrows). The yellow shaded area represents the diurnal variation of irradiance (*E*). (**b**) Changes in the PSII *t*_1/2_ in response to contrasting light exposure (in % of sea surface), fitting the data to a power function (continuous line). The dotted line indicates the relative energy cost of repairing photodamaged PSII by the symbiotic algae (*C*_a_) with light exposure, expected to be inversely proportional to the PSII *t*_1/2_. (**c**) Theoretical behavior of coral holobiont energetic performance (continuous line) in relation to *Q*_m_ (discontinuous line) across a gradient of irradiance, highlighting the predicted effects of light stress (LS) and light limitation (LL) on coral holobiont energetic performance. (**d**) Schematic representation of the relative changes in light-induced processes affecting the energy balance of coral holobionts along a depth gradient. *PUES*: photosynthetic usable energy supply; *C*_a_ energy costs of repair from photodamage for the algae; and *P*_g_: gross productivity.

The diurnal patterns of change of the photosystem II (PSII) effective quantum yield (Δ*F*/*F*_m_’) measured with pulse amplitude modulated (PAM) fluorometry, followed an inverse association with light availability (Fig. 1a). The diurnal oscillation in Δ*F*/*F*_m_’ results from the induction of photochemical (*q*_*P*_) and non-photochemical quenching (*q*_*N*_ or NPQ) processes, which occur simultaneously in the photosynthetic apparatus in order to transform part of the solar energy absorbed into organic carbon (*q*_*P*_) and dissipate the energy absorbed in excess as heat (NPQ)^23–26^. On day one in Fig. 1a, a complete recovery of the maximum quantum yield of PSII (*F*_v_/*F*_m_) was observed by the end of the day when corals were exposed to similar light conditions (control) relative to previous days (9 mol quanta m^−2^ d^−1^ with a peak irradiance of 454 μmol quanta m^−2^ s^−1^). This observation indicates that no accumulation of photodamage occurred in these corals at the end of the day as NPQ was fully relaxed. On day two, light exposure was increased by nearly three times relative to control (26 mol quanta m^−2^ d^−1^ with a peak irradiance of 1130 μmol quanta m^−2^ s^−1^) and the amplitude of the Δ*F*/*F*_m_’ oscillation was greater, reaching a minimum value at noon that was lower with respect to the previous day. This finding suggests a greater induction of NPQ processes to dissipate the increased amount of absorbed light energy. Additionally, the incomplete recovery of *F*_v_/*F*_m_ observed at dusk (0.529) compared to former dawn levels (0.643) suggests the accumulation of photodamage in the photosynthetic apparatus, potentially associated with an incomplete relaxation of the photoinhibitory quenching (*q*_*I*_) component of NPQ which requires de novo synthesis of PSII proteins (e.g., D1) for its relaxation^27–29^. The accumulation of photodamage as light exposure was increased can be explained by the rate of damage to PSII exceeding that of repair. On day three, corals were exposed to nearly half of the light levels on day one (5 mol quanta m^−2^ d^−1^ with a peak of irradiance of 237 μmol quanta m^−2^ s^−1^), which resulted in reduced oscillation in Δ*F*/*F*_m_’ and recovery in *F*_v_/*F*_m_ at the end of the day (0.625) exceeding formed dawn levels (0.523). These responses to irradiance reduction indicate a lower induction of NPQ processes and a greater relaxation of *q*_*I*_ at dusk as a result of greater rates of PSII repair compared to those of photodamage^27–29^ (Fig. 1a). The observed patterns in Δ*F*/*F*_m_’ oscillation and daily *F*_v_*/F*_m_ variation are associated with down-regulation responses of algal symbionts to protect their photosynthetic apparatus and optimize photosynthetic performance under variable light environments. Furthermore, they indicate that light exposure plays a critical role in the balance between the rates of PSII damage and subsequent repair, for which de novo synthesis of proteins for the reassembly of reaction centers is required.

To better understand the relationship between light exposure and the rates of PSII damage, a series of experiments were performed using chloramphenicol (CAP), an inhibitor of chloroplast protein synthesis that suppresses repair of damaged PSII reaction centers^30^. The difference between controls and samples exposed to CAP allowed us to determine the PSII half-time (*t*_1/2_) as a function of light exposure. The association found between PSII *t*_1/2_ and irradiance is non-linear and can be described using a power-function model (R^2^ = 0.97, *p* < 0.01) (Fig. 1b). Similar patterns have been observed in other primary producers^31,32^, suggesting that the non-linearity between PSII *t*_1/2_ and irradiance may be driven by common mechanisms across primary producers. A conceptual analysis of the changes in the maximum excitation pressure over PSII (*Q*_m_) as a function of irradiance indicates that both the shortage as well as the excess of light compromise the energetic performance of coral holobionts (Fig. 1c). This compromise results from two contrasting effects: a negligible photosynthetic activity of the zooxanthellae under light-limiting conditions (low *Q*_m_), and increased costs of repair from photodamage at high irradiance levels (high *Q*_m_)^28,33^.

The non-linearity between downwelling irradiance, photosynthetic production and energy expenditure in photorepair, leads to a unimodal, hump-shaped pattern of the photosynthetic usable energy to corals with depth (Fig. 1d). This pattern is associated with an energetic imbalance that results from the hyperbolic response of photosynthesis to light (i.e., saturation of photosynthesis above the light saturation point, *E*_k_), and the linear relationship between light availability and light absorption^34–36^. The decline in usable energy toward both ends of the depth-mediated light gradient is explained by two different processes: (1) in shallow high-light environments, the energy expenditure in photorepair increases proportionally to the amount of light above *E*_k_ while photosynthesis is saturated (*P*_max_); and (2) in deep low-light environments, the light attenuation results in a gradual decline of the photosynthetic activity. At a particular intermediate depth, where the absorbed irradiance is close to that required for maximizing photosynthesis (*E*_k_), the supply of photosynthetic usable energy and the holobiont energetic performance are predicted to reach maximum potential. The level of irradiance that is required to saturate the photosynthetic apparatus (*E*_k_) determines the threshold for the escalation of the amount of solar energy absorbed in excess relative to the maximum photosynthetic capacity (*P*_max_) of corals, which remains constant above *E*_k_.

### Photosynthetic usable energy correlates with coral biodiversity patterns

**Figure 2:**
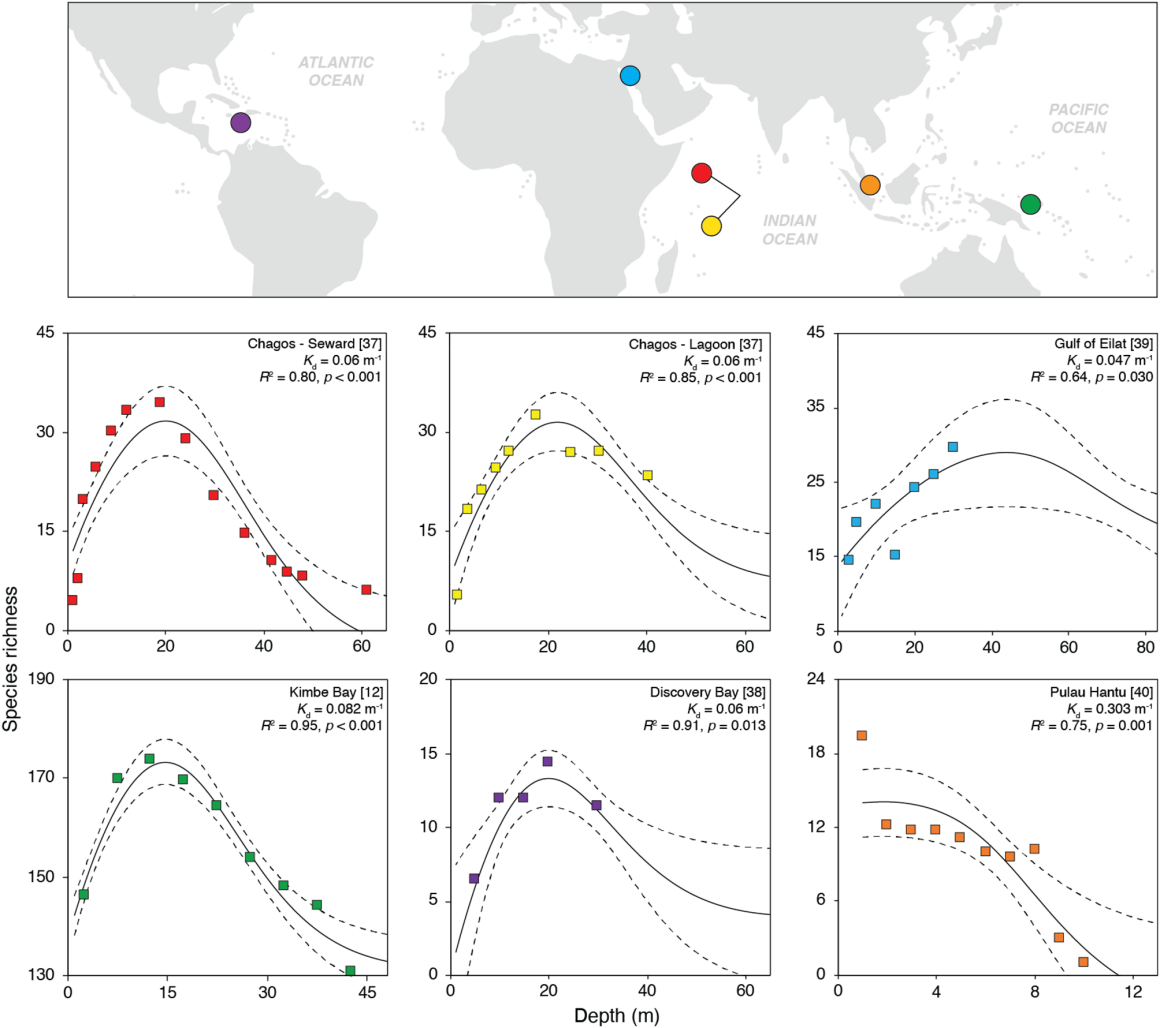
Coral species richness variation along depth gradients in reefs from major hotspots of biodiversity. The observed distribution of species richness (squares) is significantly explained by the coral productivity-biodiversity model at all sites. Continuous lines represent the trend in mean values and the discontinuous lines 95% confidence intervals. The goodness-of-fit (*R*^2^) and the statistical significance of the model (*p*-value), together with the local *K*_d_ are indicated for each site. Species richness was projected to depths at which the light intensity was estimated to be 2% of the incident irradiance at sea surface.

The coral productivity-biodiversity model explained most of the variation in coral species number with depth in all reefs tested (Fig. 2, Supplementary Tables S1, S2). The explanatory power of the model was consistently high, between 80 and 95%, in sites with unimodal relationships between species richness and depth (e.g., atolls from the Chagos Archipelago in the Indian Ocean^37^, Discovery Bay in the Caribbean^38^, and Kimbe Bay in the Indo-Pacific^10^). The model also captured the overall pattern of change in species number with depth in sites with monotonic patterns, both positive (Gulf of Eilat, Red Sea^39^) and negative (Pulau Hantu, Singapore^40^). In these sites, the model respectively explained 64% and 75% of species number variation, and in the site with a positive, monotonic relationship, it predicted a reduction in species number toward non-sampled depths below ~ 40 m, suggesting that these monotonic patterns may represent only one-half of the unimodal productivity-biodiversity relationship depending on whether the sampled depth range was located above or below the mode (Fig. 2).

**Figure 3:**
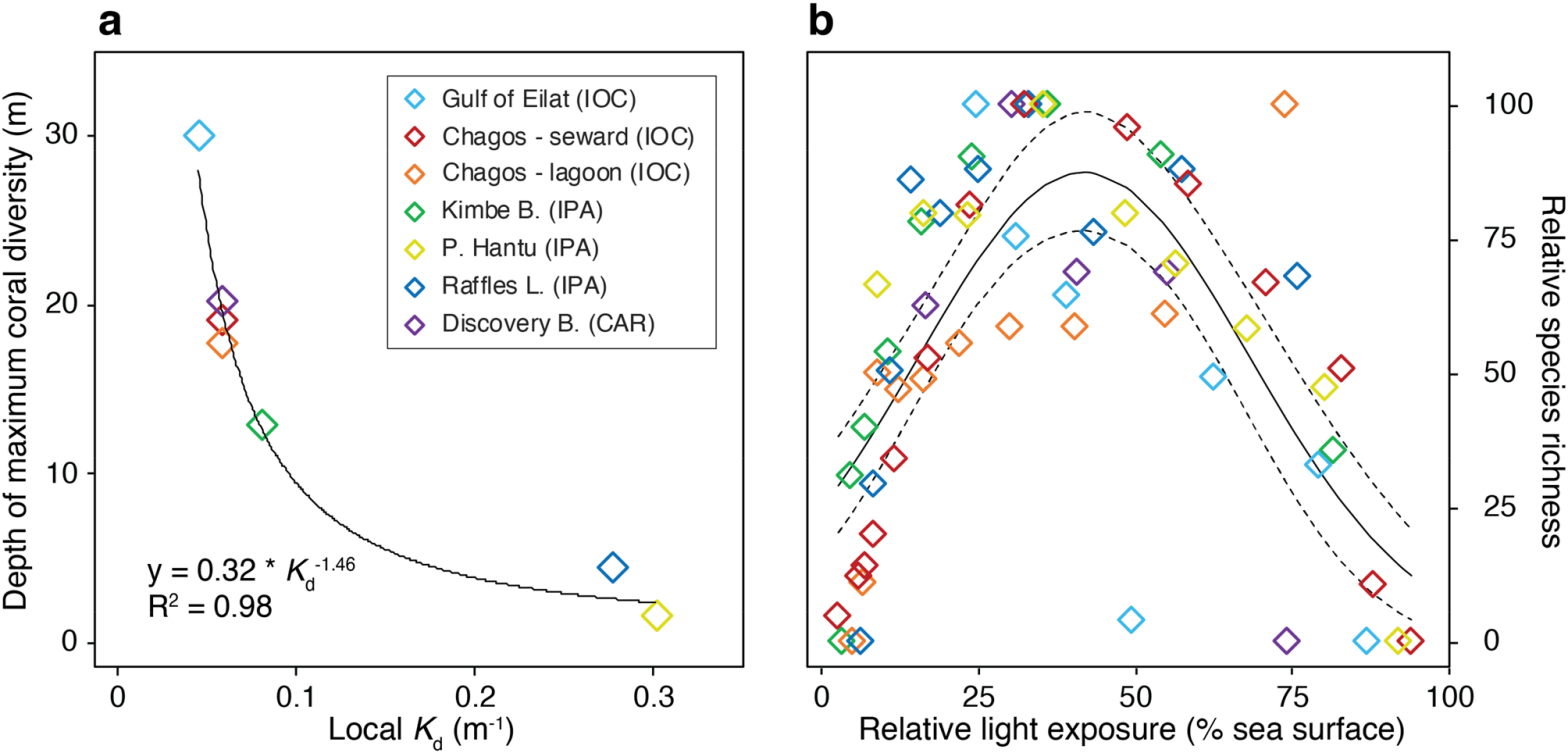
Relationship between the distribution of coral species richness and the water optical properties. (**a**) Vertical attenuation coefficient for downwelling irradiance (*K*_d_) versus the depth of maximum diversity by location. A power function depicted in the figure was used to fit a linear regression to the data. (**b**) Variation in coral species richness along a light intensity gradient determined by the local *K*_d_. Relative light exposure is reported in % of sea surface. The overall data was fitted using a gaussian function (continuous line) with 95% confidence intervals (discontinuous lines).

The *K*_d_’s in sites with unimodal relationships were consistently low (between 0.06 and 0.082 m^−1^)^10,38,41^. In contrast, in sites with positive and negative monotonic relationships, the *K*_d_’s were respectively the lowest and highest of all sites considered in our analysis (0.047 m^−1^ and 0.303 m^−1^)^40,41^ (Supplementary Table S2). The deepest record of maximum coral species richness by location was associated with the water optical properties determined by the local *K*_d_. A non-linear association between both parameters was observed and described using a power function (R^2^ = 0.98, *p* < 0.01, Fig. 3a), indicating a fast reduction of the depth of maximum coral richness with increasing *K*_d_ (Fig. 3a). The overall variation in the relative number of coral species by location along a similar light intensity gradient normalized to the percentage of surface irradiance mediated by the local *K*_d_, followed a unimodal, bell-shaped pattern with an overall reduction in the relative number of species at both ends of the light intensity gradient (Fig. 3b).

## Discussion

The repair of the photodamaged PSII reaction centers is an essential component of the photoprotection capacity of the zooxanthellae to maintain an optimal photosynthetic performance of coral holobionts in contrasting light environments^35,36^. Although photodamage and photoprotection are processes intensively studied on intact corals and/or their isolated symbionts during last decades^23–26,42–44^, the understanding of their implications on coral energetic performance at the community and ecological levels is still limited. The induction of these processes can be inferred from variations in the effective (Δ*F*/*F*_m_’) and maximum (*F*_v_/*F*_m_) quantum yield of PSII of algal symbionts *in hospite*, primarily studied by quenching analysis of the chlorophyll *a* fluorescence signal^28,35^. Two processes, photochemical (*q*_*P*_) and non-photochemical (*q*_*N*_ or NPQ) regulate the quenching of the chlorophyll *a* fluorescence signal. The first pathway, *q*_*P*_, is directly associated with the photosynthetic conversion of sunlight energy to chemical energy, whereas *q*_*N*_ or NPQ are two parameters developed in quenching analysis to quantify the photoprotective capacity of the photosynthetic apparatus to dissipate as heat the excessive excitation energy. Three main NPQ components with contrasting relaxation kinetics have been characterized: the energy-dependent quenching (*q*_*E*_), the quickest to relax associated with the operation of the xanthophyll cycle; the state-transition quenching (*q*_*T*_) associated with the redistribution of excitation energy between both photosystems, PSII and PSI, by physical modulation of their absorption cross-section; and the photoinhibition quenching (*q*_*I*_), the slowest to relax and related to the reversible photoinhibition of PSII after de novo synthesis of proteins. This last component reflects the capacity to repair inactive/photodamaged PSII^27,28^.

In this study, we parameterized the costs of repair of the photosynthetic apparatus on *in hospite* zooxanthellae as a function of irradiance. This parameterization was derived from the experimental determination of the variation of PSII half-time (*t*_1/2_) with light exposure, after suppressing the PSII repair capacity in the zooxanthellae. Differences in *F*_v_/*F*_m_ at the beginning and end of the diurnal cycle when light exposure is increased or reduced, reflect changes in the accumulation of photoinactivated PSII reaction centers. These differences arise from disparities between the rates of photodamage and repair of the photosynthetic apparatus of *in hospite* zooxanthellae in particular light environments. The kinetics of the PSII *t*_1/2_ indicates that the rate of PSII damage is proportional to light exposure, and thus the energy cost of repair the damaged PSII reaction centers for optimizing the algal photosynthetic performance should also be variable and mediated by light exposure. This assumption is supported by the evidence that a constant synthesis and replacement of proteins (e.g., D1) is required during the photosynthetic activity for the re-assembly of PSII reaction centers, and that this protein turnover can be the largest single contributor to the costs of maintenance in primary producers^45,46^. The parameterization of the energy costs to maintain photosynthesis of *in hospite* zooxanthellae along depth-mediated light gradients is a key factor taken into account in our model because of the potential limiting effect on the translocation of photosynthetic usable energy to their coral hosts, expected to have relevant ecological implications.

Bearing in mind the consistency of the productivity-biodiversity model tested here, despite local differences in reef geological history, environmental conditions and patterns of diversity, our analysis indicates that much of the variation in coral species richness along depth gradients is driven by changes in the fractional contribution of photosynthetically fixed energy by the symbiotic algae to their coral hosts. These findings support the occurrence of a productivity-biodiversity association in reef-building coral communities, similar to that widely acknowledged in communities across terrestrial environments^5,6,20^. Furthermore, this linkage highlights the fundamental role of zooxanthellae productivity in the structure of coral reef communities. The lack of prior support for a productivity-biodiversity relationship in coral communities^10^ arise from unclear definitions of the actual energy physiologically available to the coral animal (e.g., solar energy), ignoring key physiological processes that constrain the photosynthetic activity of its algal symbionts. This contradiction illustrates the difficulty of actually measuring gradients of usable energy available to organisms in studies of productivity-biodiversity associations, and of choosing the energy-related variable that best explains species richness variation according to the system studied^5–7^.

Although coral richness followed a positive relationship with the photosynthetic usable energy supply, the productivity-biodiversity association is described by a unimodal, humpbacked curve with depth whose shape and mode localization are highly influenced by the water optical properties (i.e., local *K*_d_). The overall humpbacked shape of the curve results from the non-linearity between depth, light availability, and the photosynthetic activity of the symbiotic algae. The increasing and decreasing phases of the humpbacked species-richness curve with depth result from two different processes that can limit the energetic output of the algae and, thus, the coral holobiont performance. In deep, low-light environments (the increasing phase of the richness curve), light deprivation results in a deficit of organic carbon derived from photosynthesis that can be translocated to the coral animal. In contrast, in shallow, high-light environments (the decreasing phase of the curve), the increased costs of maintenance of the photosynthetic activity once photosynthesis is fully saturated, limit the amount of photosynthetic usable energy that can be translocated to the coral host. This condition is probably coupled with a strong selective pressure exerted by intense light on the coral community in shallow habitats, including high levels of UVR^47^. At intermediate irradiance, the energetic output of the symbionts and the coral holobiont performance are predicted to reach their maximum potential, which can lead to reduced rates of species extinction and increased biodiversity^5^. The contrasting patterns observed in the productivity-biodiversity curves by location and the relationship between the depth of maximum richness and the local *K*_d_ illustrate the essential role of the water optical properties on the spatial organization and depth distribution of symbiotic coral communities. Thus, the relationship of the optical properties of the water is not only limited to the lower depth distribution of symbiotic corals^41^, but also, to patterns of coral biodiversity and community structure. It is worth noting that the water optical properties can be highly variable in some sites due to the influence of particular meteorologic and oceanographic conditions^3,48^, and that a single *K*_d_ may not represent the complex dynamics of the underwater light climate. Understanding this variability, although crucial for estimating the spatial–temporal variation in benthic primary production^4,16^, is beyond the scope of this study.

Zooxanthellate corals display contrasting photoacclimation responses, coral cover, colony morphologies and genetic richness along depth gradients^33,49–54^, which collectively suggest that coral species occupy different light niches. Particular colony geometries that optimize light capture and photosynthetic energy acquisition seem to be selected for maximizing the energy output at the colony level in specific light habitats^49,54^. Shallow-water corals, for example, adopt complex morphologies (e.g., branching and corymbose) to regulate within-colony light levels, potentially maximizing the light utilization efficiency and the photosynthetic output of the colony. In contrast, deep-water corals adopt flattened morphologies to maximize light capture and minimize self-shading^50–53^. These patterns suggest that a compromise between maximizing the photosynthetic production while minimizing the energy expenditure in photorepair may have been a driving force of colony morphology through coral evolution. Additionally, the association with symbionts with distinctive photoacclimatory potential can allow corals to cope with contrasting light climates along depth gradients and within colonies^33,55,56^. The location of maximum translocation of photosynthetic usable energy at intermediate irradiance may promote a more widely available specialization and evolutionary innovation space in that particular habitat with regard to variations in colony geometry and symbiotic associations, allowing more coral species to coexist^8^. Conversely, the variety of viable specializations that emerge from combinations between colony morphology and algal associations are predicted to be lower at both ends of the light intensity gradient due to reduced resource heterogeneity and stronger competition for resources^7^. Moreover, the reduced translocation of photosynthetic usable energy can lead to increased risk of species extinction and prevalence of few efficient competitors at exploiting the available light energy, either in excess or deficit.

Given their mixotrophic nature, heterotrophy is another aspect that has to be considered when analyzing the energy budget of zooxanthellate corals and its potential influence on biodiversity patterns across depths. Previous studies have demonstrated that some coral species are able to increase their metabolic reliance on heterotrophy to compensate for reduced photosynthetically derived energy acquisition in deep, low-light environments^57,58^, thereby potentially influencing biodiversity patterns across depths. Although we did not parameterize this aspect of the symbiosis in the documented productivity-biodiversity model, a potential effect of heterotrophic plasticity or trophic niche differentiation on coral richness cannot be discharged. However, the large component of the variability explained by the model and the contrasting features exhibited by zooxanthellate (non-facultative) corals suggest that coral adaptation and specialization to optimize solar energy utilization may have been a fundamental driving force in coral evolution, perhaps more important than enhancing heterotrophic feeding capabilities^41,52^. In this sense, if heterotrophy played a significant role in determining patterns of coral biodiversity with depth, a greater number of species with morphologies that facilitate suspension feeding (e.g., branching) would be expected to thrive with increasing depth. However, the empirical evidence indicates that flattened morphologies to maximize light capture prevail in the lower photic zone^41,52^.

Disturbance frequency and intensity have also been shown to affect the structure of coral communities, and it has been traditionally hypothesized that intermediate disturbance regimes lead to greater biodiversity^17^. The intermediate disturbance hypothesis, originally proposed as a conceptual model, has been supported and rejected in its capacity for explaining biodiversity patterns in ecological communities, both aquatic and terrestrial^7,9^. There are two major disturbances that can frequently affect shallow-water reefs, with little impact in deep-water counterparts: coral bleaching related to heat stress and high wave energy due to storms and hurricanes^15,41^. These disturbances can certainly influence both the local species diversity and the community composition of symbiotic corals^18,19^. However, the consistency of the explanatory power of the productivity-biodiversity model despite local environmental and ecological conditions among sites suggests that disturbances and other environmental factors may alter the location of the node as well as the slope of the increasing and/or decreasing phase of the unimodal curve, but not the overall pattern. Indeed, our results suggest that overlooking the role of productivity can obscure the underlying cause of biodiversity patterns in coral communities.

In summary, the results of this analysis indicate that solar energy and photosynthetic productivity are major driving forces of biodiversity patterns along depth gradients in symbiotic coral communities. The symbiosis with photosynthesizing dinoflagellates was a successful adaptive solution for corals to thrive in oligotrophic environments^11^ which, ultimately, led to the consolidation of one of the most biodiverse ecosystems on the planet. The gradient of downwelling irradiance mediated by the water optical properties, coupled with the metabolic and physiological constraints imposed by the obligate symbiosis with a primary producer, seems to be primary determinants for the establishment of global-scale patterns of biodiversity in scleractinian corals. As a major reef-building taxa, there patterns may potentially affect the associated communities that depend on corals for food and shelter. The increased worldwide degradation of the water optical properties in coastal environments associated with coastal development, nutrient enrichment, massive algal blooms, and terrestrial runoff^59–61^, may be an important underlying cause of biodiversity loss and change in the assemblage structure of coral reef communities. Local conservation actions seeking to maintain the water optical quality and the underwater light climate are essential to preserve coral biodiversity, while concerted global action to limit greenhouse emissions and slow global warming continues to move forward.

## Methods

### Zooxanthellae’ energy budgets

We assume that the energy cost of repairing the light-induced damage of the photosynthetic apparatus in the zooxanthellae is the main limiting factor for the photosynthetic usable energy supply by the algae to their coral hosts. This assumption is supported by evidence that the continual replacement of proteins required for the re-assembly of PSII reaction centers can be the largest single contributor to the costs of maintenance in primary producers^45,46^. The photosynthetic usable energy supply to corals is correlative to the “contribution of zooxanthellae carbon to the animal host respiration”, a concept originally introduced by Muscatine et al.^62^. In their original formulation, however, the translocation of photosynthetically fixed carbon was calculated based on the oxygen production/consumption of the holobiont using a factor that was independent of light availability.

We determined the pattern of change of the zooxanthellae energy expenditure in photorepair as a function of light exposure using small fragments (~ 5 × 5 cm, *n* = 50) of the Caribbean coral *Porites astreoides* collected from La Bocana Reef at 5 m depth in Puerto Morelos, México. Two weeks after acclimation in a running seawater aquarium with neutral density filters simulating the light intensity at the collection depth and controlling temperature at 28 °C, corals were exposed to contrasting light intensities to measure changes in: (1) the photosystem II (PSII) effective (Δ*F*/*F*_m_’) and maximum (*F*_v_/*F*_m_) photochemical quantum yields over diurnal cycles using pulse amplitude modulated technique^23,33^, and (2) the PSII half-time (*t*_1/2_) by analyzing the diurnal oscillations of Δ*F*/*F*_m_’ of coral fragments exposed to 100 µg of chloramphenicol (CAP), a chloroplast protein-synthesis inhibitor^30^, relative to controls without CAP.

The PSII *t*_1/2_ was calculated as the amount of time required to inactivate 50% of the PSII. We explored the optimum relationship between PSII *t*_1/2_ and light exposure (as % of sea surface) using power-function modeling. The following power function explained the variation of the PSII *t*_1/2_ with light exposure:

1 $${\text{PSII}}t_{{{1}/{2}}} = ME^{\Delta } ,$$

where *M* is the coefficient of the power function representing the maximum theoretical value of PSII *t*_1/2_, *E* is irradiance (used here to denote variation in light exposure), and *Δ* is the rate of change of PSII *t*_1/2_ with respect to available light.

The energy expenditure in photorepair by the symbiotic algae over a diurnal cycle (*C*_a_) was calculated through the relation:

2 $$C_{{\text{a}}} = \, \left( {{12 }/{\text{ PSII}}t_{{{1}/{2}}} } \right)R,$$

where *R* corresponds to the relative energy cost of protein turnover for the reassembly of PSII reaction centers over a diurnal cycle (12 h of daylight).

### Description of the coral productivity-biodiversity model

To analyze the relationship between photosynthetic usable energy and coral biodiversity across depths, we used a numerical model with a lumped-parameter approach assuming that a specific set of parameters represent the average response of the whole community^63^. The model acknowledged three physical and physiological principles that govern the vertical distribution of light in the water column and the photosynthetic activity of primary producers: (1) solar radiation diminishes exponentially with depth according to the water optical properties (i.e., local *K*_d_); (2) algal photosynthetic production describes a hyperbolic tangent response to light availability; and (3) energy expenditure for maintaining the algal photosynthetic activity is mediated by the light exposure following Eq. (2) and varies with depth according to Eq. (3). This approach allows us to draw general conclusions about light-driven processes at the community level at the expense of simplifying the complex dynamics of the water optical properties^3,16,48^, and acclimation strategies of corals to maximize performance under particular light and temperature regimes^51,64,65^.

The available solar radiation across depths, as the energy source for the symbiotic algae to fix inorganic carbon via photosynthesis, is calculated following Kirk^13^ as:

3 $$E_{z} = E_{0} {\text{e}}^{{ - K{\text{d}}z}} ,$$

where *E*_*z*_ and *E*_0_ are the irradiance (*E*) at depth *z* and just below the sea surface, respectively.

The hyperbolic response of algal photosynthesis (*P*_g_) to irradiance is calculated according to Jassby and Platt^34^, as:

4 $$P_{{\text{g}}} = P_{{{\text{max}}}} {\text{tanh }}(\alpha E_{z} /P_{{{\text{max}}}} ),$$

where α is the photosynthetic efficiency corresponding to the slope of the linear increase of photosynthesis at subsaturating irradiance and *P*_max_ is the maximum photosynthetic rates at light saturation. We assume no temporal reduction in light-saturated photosynthesis in response to photoinhibition at excessive irradiance considering the apparent absence of this phenomenon in symbiotic dinoflagellates *in hospite*^24^.

To estimate the variation of *C*_a_ as a function of depth, *C*_a_(z), we combined Eqs. (1), (2) and (3) as:

5 $$C_{{\text{a}}} \left( z \right) = \left( {{12}/\left( {{\text{M}}\cdot\left[ {E_{0} {\text{e}}^{{ - K{\text{d}}z}} } \right]^{\Delta } } \right)} \right)\cdot{\text{R}}$$

Finally, the photosynthetic usable energy supply (*PUES*) is parameterized with the following equation:

6 $$PUES = P_{{\text{g}}} - C_{{\text{a}}} \left( z \right).$$

### Testing the coral productivity-biodiversity model

We tested the explanatory power of the productivity-biodiversity model in a wide range of coral reef habitats along the world’s major hotspots of marine biodiversity (Indo-Pacific, Caribbean and Indian Ocean^66^). We analyzed published datasets from studies that reported the change in number of symbiotic coral species with depth in seven reefs with contrasting geological history, environmental conditions and biodiversity patterns (unimodal, monotonic increase and monotonic decrease) (Supplementary Table S1)^10,37–40^. Data were extracted directly from the text, tables, or original datasets. When the data was only presented in graphical format, raw values were extracted using the software WebPlotDigitizer version 4.4 (https://automeris.io/WebPlotDigitizer). When the local *K*_d_ was not reported in the manuscript, it was calculated from the light change across depths following Eq. (3). When neither of these options was possible, it was extracted from the literature for the same or comparable sites (Supplementary Table S2).

For each site, a two-step optimization procedure was used to calculate those parameters that could not be estimated independently with empirical data (*M, Δ, P*_max_, α, *R*) (Supplementary Table S2). The optimization of these parameters produced the best possible fit between the model output and the mean number of observed species across depths. First, an iterative algorithm that allows the variation of each parameter within a suspected range while fulfilling the model equations exactly was used for fitting a linear model with coral richness as a function of the photosynthetic usable energy. Using the Akaike Information Criterion (AIC), we selected the set of values that minimized the misfit between the productivity-biodiversity model output and the empirical data. Then, the values of the targeted parameters that minimized the AIC were used as the starting values in a bound-constrained minimization algorithm (*optim* function) based on the Nelder-Mead method^67^. The lower and upper bounds of each parameter were determined based on empiric data and physiological limits and were constant among sites. A diurnal cycle of solar radiation describing a 12 h sinusoidal curve peaking at 1800 μmol quanta m^−2^ s^−1^ at noon was used as forcing function (chosen maximum is a random value close to naturally occurring maximum irradiance at ocean surface in tropical areas^13^). All analyses were conducted using R version 3.6.1^68^.

## Supplementary Information

Supplementary Information 1.

Supplementary Table S1.

Supplementary Table S2.

## Data Availability

Data generated and analyzed during the current study, as well as the code used to run these analyses, are openly available on Figshare (https://figshare.com/s/b73708c98e8108ec0352).
